# Supplementary material for: Effects of brief mindfulness training on smoking cue-reactivity in tobacco use disorder: Study protocol for a randomized controlled trial
Source: PLoS One. 2024 Apr 22;19(4):e0299797. doi: 10.1371/journal.pone.0299797 (PMC11034654; doi:10.1371/journal.pone.0299797)
Supplement: S1 File — (DOCX) [file pone.0299797.s001.docx]

**S1 File. Copy of the study protocol approved by the ethics committee**

**Effects of brief mindfulness training on smoking cue-reactivity in tobacco use disorder: Study protocol**

1. ***Declaration of Integrity. [including two aspects: (1) guaranteeing that the operation strictly follows the experimental procedures and the authenticity of data records; (2) truthfully disclosing any conflicts of interest]***

The research will ensure that all operations are strictly follow the trial procedures and authenticity of data records; There are no interests conflicts in this study.

1. ***Title. [The research title should accurately reflect the research purpose, be concise and brief, and adhere to the PICOS structure, which includes the disease or research subject, treatment methods, control methods, outcomes or research objectives, and research or design type. （participants, intervention, control, outcome, study）]***

Effects of brief mindfulness training on smoking cue-reactivity in tobacco use disorder

1. ***Protocol version (Date and version identifier)***

V1.0

1. ***Funding (Sources and types of financial, material, and other support)***

National Natural Science Foundation of China (No. 32060196)

1. ***Flowchart or table of the research implementation process, with SPIRIT template attached. [arranging tasks to be performed at each time point of the study].***


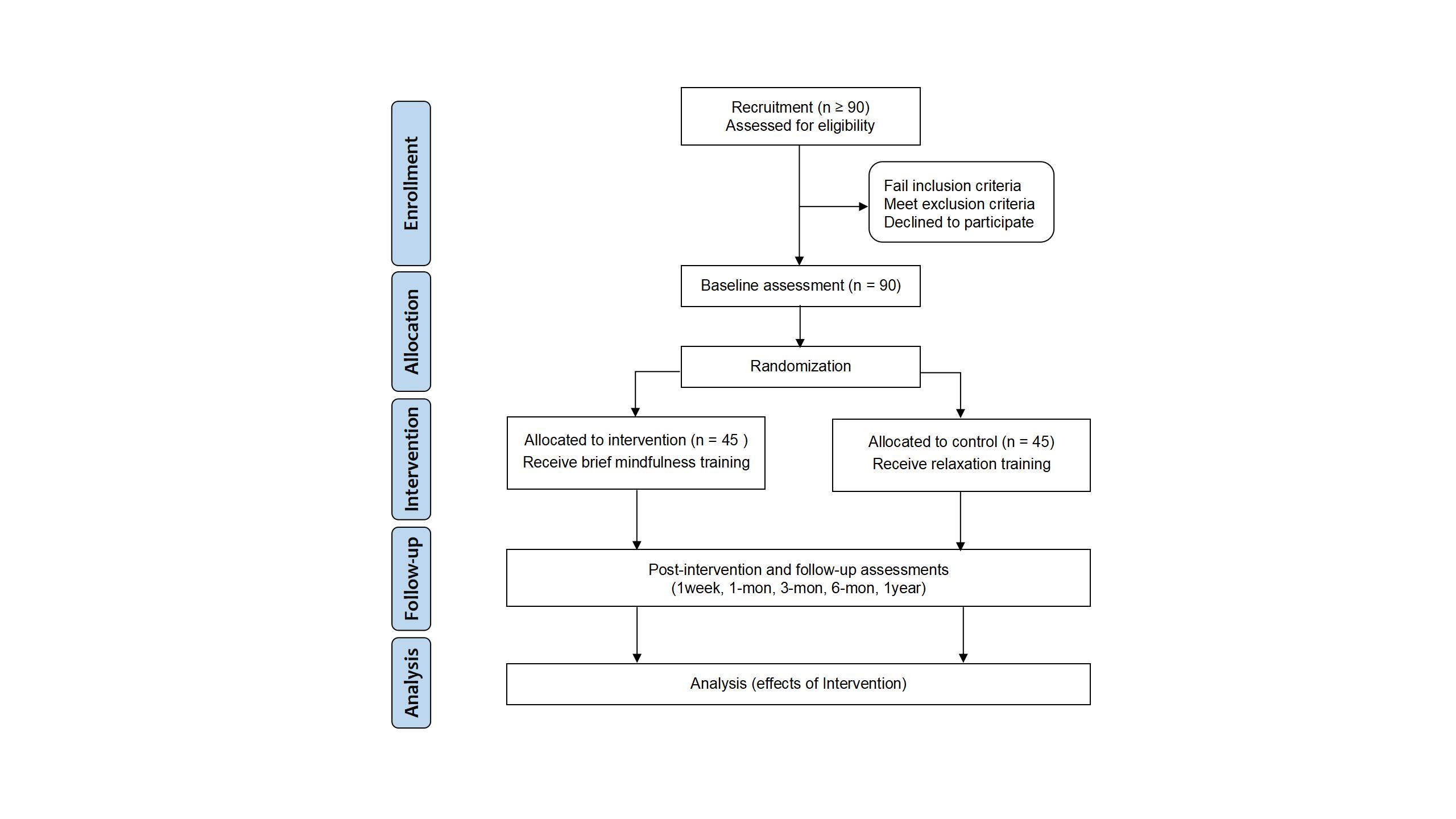


**Fig 1. SPIRIT Flowchart**

|  | **STUDY PERIOD** | | | | | | |
| --- | --- | --- | --- | --- | --- | --- | --- |
|  | **Enrolment** | **Allocation** | **Post-allocation** | | | | **Close-out** |
| **TIMEPOINT*** | ***-t_1_*** | **0** | ***t_1_*** | ***t_2_*** | ***t_3_*** | ***t_4_*** | ***t_5_*** |
| **ENROLMENT:** |  |  |  |  |  |  |  |
| **Eligibility screen** | X |  |  |  |  |  |  |
| **Informed consent** | X |  |  |  |  |  |  |
| **Baseline assessment** | X | X |  |  |  |  |  |
| **Allocation** |  | X |  |  |  |  |  |
| **INTERVENTIONS:** |  |  |  |  |  |  |  |
| ***[Brief mindfulness training]*** |  |  |  |  |  |  |  |
| ***[Relaxation training]*** |  |  |  |  |  |  |  |
| **ASSESSMENTS:** |  |  |  |  |  |  |  |
| ***[Demographic data]*** | X |  |  |  |  |  |  |
| ***[Primary outcome variables]*** | X | X | X | X | X | X | X |
| ***[Secondary outcome variables]*** | X | X | X | X | X | X | X |
| ***[Daily records during intervention]*** |  | X | X |  |  |  |  |

**Fig 2. SPIRIT schedule of enrolment, interventions and assessment.**

** Note: -t1, 0, baseline; t1, post intervention; t2-t5, 6-week, 1-month, 6-month, 1-year follow-up.*

1. ***Research background. [usually adopting a four-part structure: (1) Overview of the health problem or research question: definition of the health problem, epidemiological data, etiology, natural history of the disease, disease burden, etc.; (2) Scientific hypothesis: mechanism or principle; (3) Clinical application, for example, treatment studies should explain the treatment methods; (4) Necessity of the study based on current evidence, explaining why this study needs to be conducted]***

Tobacco Use Disorder (TUD) is a pressing public health concern in China **^[1]^**, posing a significant threat to the overall well-being, and being a leading cause of death and disability of the population **^[2-5]^**. Understanding the physiological and subjective responses, such as cravings and impulses triggered by smoking cues, is crucial for comprehending the development and maintenance of addiction **^[6, 7]^**. Previous research has demonstrated that mindfulness training can influence conditioned response behaviours **^[8]^**, including reducing classical conditioning, delaying primary responses, and decreasing the frequency. Therefore, mindfulness has been widely utilized as an effective treatment for addiction and relapse prevention **^[9]^**, as it reduces subjective cravings, attentional focus, and physiological indicators associated with substance cues. By targeting negative emotions and somatic sensations, mindfulness can dissociate emotional responses from cigarette cravings **^[10]^**.

Nevertheless, the extended time commitment and the need for in-person guidance in traditional mindfulness interventions have resulted in a growing need for shorter, self-practice exercises. According to reports, even single-session mindfulness interventions and brief interventions lasting as little as 5 minutes have shown improvements in health outcomes **^[11, 12]^**. Brief mindfulness interventions have been found to reduce smokers' response to cravings **^[13]^**, improve emotional health **^[14]^**, decrease smoking consumption **^[15]^**, and have been associated with higher rates of abstinence **^[16]^**. Study in natural situation has also demonstrated that brief mindfulness training (BMT) can decrease self-reported negative emotions, smoking cravings, and cigarette consumption **^[17]^**, indicating its effectiveness in smoking cessation treatment.

Furthermore, mindfulness can influence interoceptive processing, potentially regulating brain activity related to cue-reactivity **^[9]^**. Previous research has observed functional changes in interoceptive brain regions **^[18]^**, such as the insula **^[19]^**, as well as control systems including the cingulate gyrus, parietal lobe, and prefrontal cortex **^[20, 21]^** following short-term mindfulness practice. However, there is currently a lack of in-depth research on the regulation of smoking cue responses and related brain mechanisms by brief mindfulness interventions in individuals with TUD.

Therefore, this study aims to explore the intervention effects of BMT on smoking cue-reactivity using a randomized controlled trial design. It seeks to address the limitations of long-duration mindfulness interventions with low acceptance rates among individuals with TUD and provide more targeted intervention strategies by investigating the underlying brain mechanisms. The results of this study will offer valuable insights that can enhance our understanding and support the clinical implementation of short-term mindfulness interventions in addiction treatment.

1. ***Objective***

This study aims to investigate the regulatory effects of BMT on smoking cue-reactivity by integrating subjective reports of smoking cravings with electroencephalography (EEG) data analysis. Additionally, it is dedicated to enhancing our understanding of the underlying brain processes involved and identifying potential EEG biomarkers at various timepoints.

1. ***Inclusion and exclusion criteria. [Inclusion criteria define the main body of the study and determine the representativeness of the study population; exclusion criteria define individuals within the study population that may affect the study and determine the homogeneity of the study population]***

Inclusion criteria (1) Right-handed. (2) Smoking an average of 10 or more cigarettes per day (half a pack of cigarettes). (3) Smoking history of at least 1 year. (4) Exhaled carbon monoxide (CO) levels detected by instruments are between 10-7ppm or lower (abstinent from smoking cigarette for 3 h prior to every visit). (5) Age between 18 and 40 years old. (6) Normal vision or corrected to normal vision. (7) Normal mental and physical health conditions (PHQ-9 **^[22-24]^** total score <20, GAD-7 **^[25, 26]^** total score <11). (8) Meet the diagnostic criteria for tobacco use disorder in DSM-5 (through clinical semi-structured interviews **^[27]^**).

Exclusion criteria: (1) Asthma, contact dermatitis, or allergies to silicone. (2) Recent use of corticosteroid medications within the past 3 months. (3) Practiced any meditation, yoga, tai chi, or qigong for more than 20 hours in the past year or lifetime, participated in meditation or yoga retreats, and attended any meditation courses. (4) Not suitable for EEG (e.g., metal implants, severe head trauma and electrode allergy). (5) PHQ-9 total score ≥20. (6) GAD-7 total score ≥11. (7) Adhere to specific religious beliefs and therefore unable to participate in meditation as required by the program. (8) Currently participating in similar trials or other neurophysiological experiments.

1. ***Study design, design pattern diagram, with SPIRIT template attached. [Clearly state the design scheme used in this study, such as: consecutive case or single-arm design, cross-sectional study, case-control study, cohort study, including historical cohort study or prospective cohort study, nested case-control study, randomized controlled trial, clearly specify the type, such as parallel design, crossover design, factorial design, single-group design; for treatment studies, specify the type of research objective framework, such as superiority, equivalence, non-inferiority, exploratory]***

To examine the effectiveness of brief mindfulness intervention in reducing smoking cravings, this study employs a randomized controlled parallel trial design. Expanding upon existing literature that supports the positive effects of brief mindfulness interventions, the primary aim of this study is to establish the superiority of brief mindfulness intervention over the control group within the intervention conditions. The trial consists of four distinct stages: participant recruitment, baseline measurement, measurement during the intervention period, and follow-up measurement. After the completion of each stage, pertinent evaluations will be conducted to assess the outcomes. The design flowchart, depicted in **Fig 3.**, illustrates the methodology employed in this study. It is important to note that the trial has not yet begun, and it is expected that the entire process, including participant recruitment, data analysis, and article writing, will take approximately one year. Additionally, a follow-up period of one year will be conducted to assess the long-term effects of the intervention.

**
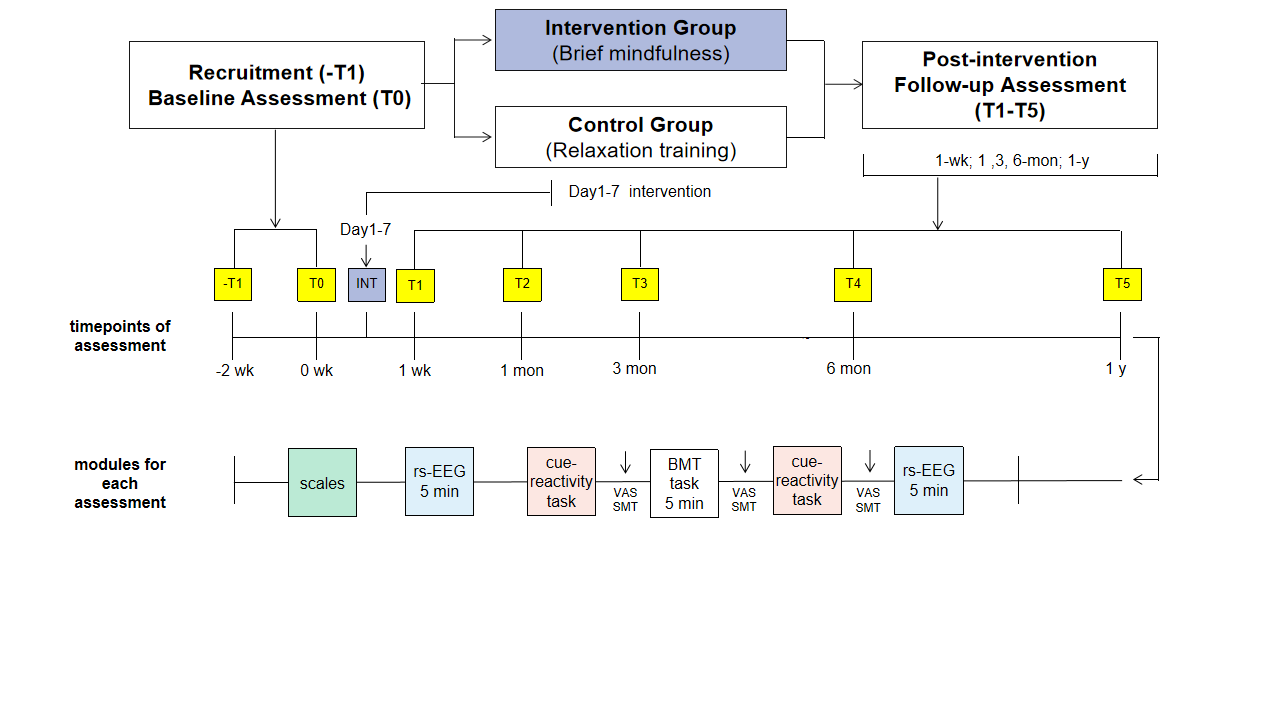
**

**Fig 3. Study Design and Procedure**

*The upper portion of the figure illustrates the overall study design. The middle portion depicts the timeline for assessing scales and EEG at baseline, post-intervention, and follow-up timepoints. Brief mindfulness and relaxation training will be administered to both the intervention and control groups after recruitment. The baseline assessment (-T1, T0) will be completed before the intervention. Audio guidance will be provided for daily practice in the BMT group, while there will be no additional practice or intervention for the control group after training. The lower portion of the figure represents the modules of psychological and behavioural assessments during the baseline and five follow-up sessions. The cue-reactivity task will be implemented using a customized two-choice oddball paradigm. INT: intervention, rs-EEG: resting-state EEG; BMT: brief mindfulness training; VAS: Visual analogue scale; SMT: state-mindfulness.*

1. ***Sample size estimation. [List the four elements: δ(Δ) or π1, π2, α, β or Power, and provide the calculation formula]***

In this study, the subjective reports of smoking urges (QSU-Brief) will be used as the primary outcome measure. Referring to study of similar populations **^[28]^**, We set δ(Δ)_tot_ = 4 to represent the anticipated reduction of 4 points in the QSU-Brief score after the intervention in the experimental group. Multiple measurements of the QSU-Brief will be conducted on participants before and after the training, as well as during the 60-minute online training period. To estimate Cohens’d, we have set α = 0.05 and β = 0.80. Cohens’d can be calculated using the formula Cohens’d=(δ(Δ)/SD_estimate_), where the estimated SD_estimate_ was 7.65 based on reference study. Therefore, the corresponding command is pwr.t.test (d=cohens_d, sig.level = 0.05, power = 0.80, type = "paired", alternative = "two.sided"), and the estimated sample size is 34. Therefore, the minimum required sample size for the experimental group is 34. Considering a natural dropout rate of 30%, the sample size for the experimental group should be no less than 45.

1. ***Methods of randomization and concealed allocation. [Randomization is used to reduce the risk of selection bias. Methods for generating random number sequences include computer software, random number tables, semi-random or pseudo-random methods (such as using dates, birthdays, or even/odd numbers of hospital visits). Please describe the individuals involved in generating random number sequences and the methods they use. Concealed allocation is used in treatment studies to reduce the risk of selection bias. Key steps in concealed allocation include having a designated person determine the allocation sequence, ensuring that this person is not involved in enrolling participants, and securely storing the allocation table. Please provide detailed descriptions of these steps. Central randomization is a robust method of concealed allocation. Describe the individuals and methods involved in determining the allocation sequence]***

(1) In this study, the intervention period for the BMT, which is a behavioural training, lasts for one week. Baseline data, including EEG data, will be collected from all participants. Following this, there will be a one-week training period for the intervention content, after which post-intervention data, including scales, self-reports, and EEG data, will be collected. Follow-up measurements will be conducted at 1 week, 1 month, 3 months, 6 months, and 1 year after the trial's completion. Group allocation will be performed after the completion of baseline data collection.

(2) The primary intervention method employed in this study is the brief mindfulness-based intervention of "STOP" **^[29, 30]^**, with a 7-day ~60-min sessions mindfulness course for the experimental group. Meanwhile, the control group will undergo relaxation training. All recruited participants are from the same school, and due to the lack of blinding execution conditions, achieving double-blind standards may present challenges.

(3) The randomization process for this trial will involve the use of a random sequence generator website (https://www.random.org/). This website will be utilized to ensure the random assignment of participants to either the experimental or control group.

1. ***Blinding. [Blinding is used in treatment studies and diagnostic trials to reduce the risk of implementation bias and measurement bias. Implementing strict confidentiality measures, such as using placebos or simulating procedures, is an effective blinding method. If blinding is necessary and feasible for your study, describe the methods and procedures for implementing blinding]***

This trial includes group therapy sessions for students from the same school. As a result, participants may already know each other or be unfamiliar with one another, and they may naturally discuss the experimental training they receive. Maintaining blinding for the researchers is challenging as they may be influenced by the experimental content during the trial. Therefore, meeting the requirements of double-blinding in a clinical trial is not feasible in this situation

1. ***Outcome Measures. [Outcome measures are used to evaluate the results of a study and can include primary, secondary, and safety measures, including adverse events and reactions. Please describe the names of the measures, the methods of measurement, and the time points at which they are assessed]***

As listed in **Table 1**, the primary outcomes include subjective reports of smoking craving, changes in EEG indicators, and mindfulness measures (both trait- and state- mindfulness). The secondary outcomes will be daily smoking behaviours (number of cigarettes smoked per day, exhaled carbon monoxide concentration), affect and impulsivity, as well as indicators reflecting correlation between mindfulness and smoking cue-reactivity. Researchers will inquire daily during the intervention period whether participants experience tobacco withdrawal symptoms or any other adverse reactions. Participants are also encouraged to proactively contact the research team if they experience any adverse reactions. Please refer to item 9 for the time points of assessment.

| **Table 1. Evaluated variables** | | | |
| --- | --- | --- | --- |
| **Variables** | **Timepoints** | **Measures** | **Outcome level** |
| **Category 1: smoking related indices** | | | |
| **Nicotine dependence** | -T1, T0, T1-T5 | **Fagerstrőm test for nicotine dependence (FTND)** is a six-item revision of the Fagerstrőm Tolerance Questionnaire **^[31]^**，scored as 0-3 (low), 4-6 (moderate), and 7-10 (high) with the summed score **^[32]^**. | primary |
| **Craving** | -T1, T0, T1-T5 | **1.Tobacco Craving Questionnaire-Short Form (TCQ-SF**）is short form of 12 optimized projects to assess smoking craving levels，including four factors of emotionality, expectancy, compulsivity, and purposefulness **^[33]^**, with each factor scores from 3 to 21 points, was used in Chinese population**^[34]^**. | primary |
|  |  | **2.Questionnaire on Smoking Urges-Brief（QSU-Brief）**developed by Cox et al., **^[35]^** suitable for multiple measurements in laboratory and clinical use, including 10 items, can be completed in less than 2 minutes. Each item uses a subjective score of 1-7 points to evaluate the current level of craving for tobacco, the Chinese version will be adopted **^[36]^**. |  |
| **VAS for craving** | -T1, T0, T1-T5, during CR task | **Visual analogue scale (VAS)** administered during cue-reactivity experiment to measure the state of craving, responses to each item were rated using a scale that ranged from 0 (not at all) to 10 (the strongest feeling possible). | primary |
| **Number of cigarettes smoked per day** | -T1, T0, T1-T5 | Self-report for number of cigarettes smoked per day. | secondary |
| **Exhaled carbon monoxide (CO)** | T0, T1-T5 | Instantaneous reading device (Bedfont piCO Smokerlyzer) used to identify whether the duration of cigarette cessation meets the requirements. | secondary |
| **Category 2: mindfulness** | | | |
| **Trait-mindfulness** | T0, T1-T5 | **Five Facet Mindfulness Questionnaire（FFMQ）**consisting of 39 items and 5 factors, namely observation, description, acting with awareness, non-reactivity, and non-judging, the Chinese version will be used**^[37]^**. | primary |
| **State-mindfulness** | T0, T1-T5, during CR task | Based on the Toronto Mindfulness Scale (TMS), two items in Chinese version **^[38]^** were used to evaluate the dimensions of curiosity and decentralization. | primary |
| **Category 3: brain function** | | | |
| **Brain function** | T0, T1-T5 | Using a 64-channel EEG system to record in both resting and experimental conditions. | primary |
| **Category 4: Socio-demographic and clinical information** | | | |
| **Self-emotional assessment** | T0, T1 | **Self-assessment manikin (SAM)** used to measure the emotional state during 7-day mindfulness intervention. | others |
| **Affect** | -T1, T0, T1-T5 | **Positive And Negative Affect Scale（PANAS）**compiled by Watson et al. **^[39]^**, Chinese version **^[40]^** includes 10 corresponding adjectives that described either positive or negative emotion in both the both the negative and positive affect scales. Each project scores 1-5 points, with a total score of 10-50 points. to measure the emotional feelings of the past week. | secondary |
| **VAS for affect** | -T1, T0, T1-T5, during CR task | **Visual analogue scale (VAS)** for affect administered during cue-reactivity experiment to measure the state of affect, including valence and arousal. | secondary |
| **Impulsiveness** | -T1, T0, T1-T5 | **The Barratt Impulsiveness Scale Version 11 (BIS-11)** **^[41]^** was revised in 1995, consisting of a total of 30 items. The revised Chinese version **^[42]^** consists of 30 items, including three dimensions: motor impulsiveness, cognitive impulsiveness, and no planning impulsiveness, with a total score of 30-150, High scores represent hyperactivity, lack of concentration, and lack of planning, respectively. The higher the factor scores of each subscale, the stronger the impulsivity. | secondary |
| **Record of daily practice for mindfulness** | T0, T1 | Self-report of practice time; cognitive, emotional and behavioural states; course feedback and suggestions. | others |
| **Adverse events** | T0, T1-T5 | Proactive reporting without time constraints during research period. | others |
| **Socio-demographic** | -T1 | Age, gender, major, education year, Handedness | others |
| **Handedness** | -T1 | **Edingburgh Handedness Inventory（EHI）**is a measurement scale used to assess the dominance of a person’s right or left hand in everyday activities, sometimes referred to as laterality **^[43]^**, the Chinese version will be used**^[44]^**. | others |
| **Depression** | -T1 | **Patient Health Questionnaire (PHQ-9)** **^[23]^** self-administered tools for assessing depression for recent two weeks, with each project scoring 0-3 points and a total score of 0-27 points, with 6-9 points, 10-14 points, 15-21 points and 22-27 points indicating mild, moderate, severe and extreme severe depression. | others |
| **Anxiety** | -T1 | **Generalized Anxiety Disorder (GAD-7)** used to measure anxiety related problems over the past two weeks, with each project scoring 0-3 points and a total score of 0-21 points **^[25]^**, with scores of 5, 10, and 15 corresponding to the cutoff values for "mild", "moderate", and "severe" anxiety levels **^[45]^**. | others |

*CR: cue-reactivity.

1. ***Definition of Participant Effectiveness Determination. [This includes definitions for participant withdrawal, exclusion, loss to follow-up, confounding factors, study discontinuation, and study suspension]***

(1) **Participant Withdrawal:** Each participant has the right to withdraw from the study at any time without the need to provide a reason. Participants who choose to withdraw voluntarily will not be included in the analysis. As part of our comprehensive research procedure, we have established a protocol for managing participants who decide to withdraw. If participants are willing to provide a reason for their withdrawal, it will be documented and recorded.

(2) **Participant Exclusion:** Due to the inability to ensure full intervention participation in the intervention group during the training process, the "STOP" training period lasts for one week. Participants who have participated in less than 5 training sessions will be considered as not having received the complete intervention and, theoretically, should be excluded or adjusted using mixed effects models.

(3) **Participant Loss to Follow-up:** If a participant cannot be contacted for two consecutive visits after the completion of the intervention for any reason, they will be classified as lost to follow-up.

(4) **Participant Contamination:** Following the completion of the intervention, a survey will be conducted with the participants. If the control group attempts to learn about the training mode used by the intervention group (or vice versa) and attempts to replicate the training, they will be classified as contaminated participants.

(5) **Participant Termination:** To prioritize participant health, any adverse events (including tobacco withdrawal symptoms) will be considered termination criteria. Researchers will discuss with the participant who experiencing such conditions and terminate the intervention early. Participants will receive appropriate clinical interventions only with their explicit consent.

(6) **Participant Suspension:** Participants in this study can request to suspend the intervention. If a suspension is requested, the participant will be evaluated by the study team and, if approved, moved to the future intervention group. Data collected before the suspension will not be included in the final data analysis.

1. ***Definition, identification methods, and management systems for adverse events and reactions.***

In the "Chinese Clinical Smoking Cessation Guidelines," it is stated that smokers may experience a range of withdrawal symptoms after quitting or reducing smoking. These symptoms include smoking cravings, anxiety, depression, restlessness, headache, increased salivary gland secretion, lack of concentration, and sleep disorders. Withdrawal symptoms typically begin within hours of smoking cessation, peak within the first 14 days, and gradually diminish until they disappear. Most withdrawal symptoms last for about one month, although some patients may experience cravings for smoking for over a year.

**Definition:** Adverse events refer to any unfavorable or unintended signs, symptoms, or diseases that occur during or after the intervention, regardless of whether they are related to the intervention. Adverse reactions specifically refer to unfavorable or unintended responses that are causally related to the intervention.

**Identification Methods:** Adverse events and adverse reactions can be identified through various methods, including participant self-reporting, researcher observations, physical examinations, laboratory tests, and medical records.

**Management System:** In light of the potential adverse reactions mentioned above, the researchers will conduct daily inquiries during the intervention to determine whether participants have experienced any of the symptom. The delivered list will also provide information to participants, instructing them to promptly contact the researchers if they experience similar symptoms at any other time. In the event of any adverse reactions, responsible personnel from the medical school and psychology department will promptly contact the participants and assess their psychological and physiological conditions. Based on the participant's willingness, a collective decision will be made by the medical school, psychology department, and participant regarding their continued participation in the subsequent research. The researchers will record the reasons for their decision.

Regarding adverse reactions during EEG data collection, if participants experience physiological rejection responses or resistance emotions, the researchers will immediately halt the EEG data collection. Whether to continue the experiment or not will be determined based on the participant's own willingness, ensuring their health and safety. The researchers will record the reasons for their decision. The subsequent follow-up and intervention procedures of this study will be communicated to the participants after collective discussion among the research team, and decisions will be made accordingly.

1. ***Ethical considerations. [This includes the selection of an ethics committee, approval procedures, informed consent process, registration time, and registration authority (please provide registration number after completion)]***

(1) **Selection of Ethical Committee:** The chosen ethical committee for this trial is the Medical Ethics Committee of Kunming University of Science and Technology, which is located within the institution where the trial is being conducted.

(2) **Approval Process:** Before initiating the study, the research plan, project proposal, and other pertinent documents underwent submission for approval in accordance with the required procedures of the designated ethical review committee at the research institution.

(3) **Informed Consent Process:** Prior to the initiation of the study, researchers furnish participants and authorized representatives with a printed version of the informed consent form. They also clarify any inquiries pertaining to the informed consent form, ensuring that participants and authorized representatives possess a comprehensive understanding of the content before proceeding to sign the informed consent form.

(4) **Registration Time:** The trial will be registered with the China Clinical Trial Registry after receiving approval from the Ethical Committee.

1. ***Participant recruitment. [This includes the selection of recruitment locations, recruitment methods, the screening process, and the researchers conducting the screening]***

Dedicated personnel are responsible for collecting general information from participants. The general information collected includes the following details: name, gender, age, grade, major, ethnicity, handedness, years of smoking, average number of cigarettes smoked per day, current participation in similar trials, and physical and mental health status, among other relevant factors.

1. ***Collection of general participant information. [This includes the researchers responsible for data collection and the content of the general participant information]***

(1) Recruitment Location: The recruitment will be conducted in a designated group training room located on campus.

(2) Recruitment Methods: This study will employ a combination of online and offline methods for participant recruitment. Offline methods include delivering lectures in classrooms, creating posters, distributing flyers, and employing other similar approaches. Online methods involve utilizing platforms such as Qzone, WeChat Moments, as well as campus-specific platforms like the campus BBS and school's bulletin board.

(3) Screening Process: Participants will be selected in the order of their participation and based on their compliance with the inclusion and exclusion criteria.

(4) The screening process will be performed by dedicated research personnel assigned specifically for this purpose.

1. ***Baseline measures and observed variables. [For treatment studies, observed variables may not necessarily be efficacy evaluation measures, such as height and weight, but may be related to drug dosage without direct relevance to efficacy evaluation]***

(1) **Baseline Indicators:** The baseline indicators for this study include demographic information (name, student ID, gender, age, major, ethnicity, contact information); handedness; history of mental illness or psychiatric disorders; Patient Health Questionnaire (PHQ) and Generalized Anxiety Disorder (GAD) assessment; years of smoking, average number of cigarettes smoked per day; measures of craving and smoking related behaviour, access by using QSU-Brief (Questionnaire of Smoking Urges-Brief) and Nicotine Dependency Test Scale (FTND); exhaled carbon monoxide concentration; mindfulness measures (both trait- and state- mindfulness); affect measured by PANAS (Positive and Negative Affect Schedule): VAS (Visual Analog Scale) for craving of cigarettes and emotions/affection. Impulsivity measured by using Barratt Impulsiveness Scale; brain function in pre- and post-intervention period.

(2) **Observed variables:** The measurement tools and specific content are shown in **Table 1**. The outcome level and timepoints for assessment was annotated.

1. ***Standard operating procedures. [For treatment procedures, treatment methods, treatment courses, etc.; diagnostic trial standard procedures; etiological research standard procedures; prognosis research standard procedures; epidemiological research standard procedures]***

Trained personnel rigorously adhere to the main operating procedures related to this research process. The standard operating procedures are as follows:

(1) **Procedure for EEG data collection:**

- 1. Inform the participants of relevant precautions before collecting EEG data and prepare for data collection (including software and hardware). Software refers to the professional software for connecting EEG devices to computers, and hardware refers to the necessary auxiliary devices such as EEG caps and conductive paste.
  2. Measure the head circumference of the participants to determine the size of the EEG cap to be worn (large cap: 56-60cm, small cap: 54-58cm).
  3. If the participants have long hair, tie it up with a headband before wearing the EEG cap to expose both ears (participants can prepare their own headbands, or they can be provided by the laboratory).
  4. Wear the EEG cap correctly on the participant's head according to the front and back of the cap and the electrode positions (the side with letters facing up, with the letter "F" indicating the front and the letter "O" indicating the back).
  5. Measure the distance from the nasion to the inion and adjust the EEG cap back and forth to ensure that the Cz electrode is at the center of this distance. Measure the distance from the left ear lobe to the right ear lobe and adjust the EEG cap left and right to ensure that the Cz electrode is at the center of this distance. The Cz electrode should be at the intersection point of the two distances.
  6. Visually inspect the participant from the front to ensure that the EEG cap is not rotating or loosening. If there is looseness or incorrect electrode positioning, repeat step 5 until the EEG cap is properly worn.
  7. Use a special syringe (bent-tip syringe) to apply conductive paste to the corresponding channels on the scalp. Since the hair needs to be moved aside with the syringe, ask the participant about the strength of application during the process and adjust accordingly based on their response.
  8. Insert the active electrodes with labeled channels into the corresponding electrode names on the EEG cap (be careful to match the labels of the active electrodes with the EEG cap). Wrap the CMS and DRL active electrode wires around the cables of the other electrodes (wrap them 3-5 times), and plug their connectors into the corresponding ports of the amplifier.
  9. After confirming that the EEG software and hardware are properly connected, run the software for collecting EEG data. Follow the instructions of the software and confirm that the quality of the EEG data meets the standards before starting the data collection. Save the EEG data to the computer or hard drive according to the naming format.

(2) **Procedure for brief mindfulness training:** The researchers of this experiment will collaborate with professional psychology teachers for a one-week training on intervention content. Prior to each practice session, participants will be reminded of the training's schedule and location, and researchers will be present to sign in on site. The one-week training on intervention content will be conducted by professional teachers from the Students Counseling and Mental Health Center at Kunming University of Science and Technology. The training will be led by experienced psychology teachers, and the content for each day's practice will be shared with participants through their group chat for continued practice after each session.

1. ***Statistical analysis methods. [Select appropriate statistical analysis methods based on the data type of each measurement variable. Include any additional methods for subgroup analysis or auxiliary analysis. If there are personnel reductions during the trial, indicate whether an intention-to-treat (ITT) analysis was used and compare it with per-protocol (PP) analysis]***

All data will be analyzed upon completion of data collection. The data analysis process will strictly adhere to the principles of the blind method. Analysis of the data will be conducted using R software (https://www.r-project.org/), IBM SPSS Statistics for Windows, V20.0 (SPSS), MATLAB (MathWorks in Natick, Massachusetts, USA), and EEGlab (https://sccn.ucsd.edu/eeglab). Descriptive statistics will be used for demographic, psychological, and behavioural data at baseline. Two-sample t-tests and χ^2^ tests will be performed for continuous and categorical variables between the intervention group and control group. Repeated measures MANOVA will be adopted to examine intervention effects while adjusting for individual factors and pre-intervention variables (e.g., trait mindfulness and smoking indices). Within-group effects will be investigated through serial trend analysis from -T1 to T5. Linear mixed-effects model analysis will include data from all measures at different timepoints to explore factors associated with smoking cue-reactivity for both groups. EEG data will be preprocessed using Matlab software and the EEGLab toolbox. Relative spectrum power for six EEG bands (delta: 1-4Hz, theta: 4-8Hz, alpha: 8-13Hz, low-beta: 13-20Hz, high-beta: 20-30Hz, and low-gamma: 30-48Hz) will be calculated at the single electrode and ROI level. Microstate and phase synchronization analysis will be conducted, and source localization analysis will be used to investigate functional changes in subcortical brain regions. Repeated measures analysis of variance and simple effect analysis will be performed for EEG indices, considering significance in the professional field. Effect size will be measured using Cohen's d or partial eta-squared values **^[46]^**.

1. ***Participant management system.***

(1) **Contact and Guidance:** A contact group consisting of relevant researchers is responsible for providing guidance and addressing any study-related questions for participants throughout the entire study period. This ensures that participants have a clear understanding of the study and can actively cooperate with the research process. Researchers are required to respond promptly and keep records for participants who withdraw, are excluded, lost to follow-up, terminated, or suspended.

(2) **Emergency Team:** The emergency team comprises researchers and psychological teachers from the Students Counseling and Mental Health Center at the school. This team promptly identifies and addresses any participants who experience adverse reactions during the study. In cases where adverse reactions cannot be resolved, the team offers additional support and solutions to assist participants. Participants are encouraged and expected to proactively reach out to members of the emergency team if they encounter any health-related issues at any time.

(3) **Confidentiality of Information:** Any information and data obtained about participants throughout the entire study will be treated with the utmost confidentiality. Unless participants give explicit permission, no information that could identify them will be shared with individuals outside the research team. Public reports on the study results will not disclose the personal identities of the participants.

1. ***Specimen management system. [This includes the processes and systems for specimen collection, storage, and submission]***

In this experiment, the concentration of exhaled carbon monoxide (CO) is measured in parts per million (ppm) and utilized as an indicator of smoking cessation duration. The Bedfont pi CO+Smokerlyzer, a portable cigarette sensor is used to assess participants' compliance with cigarette abstinence criteria. The data collection procedure is similar to measuring breath alcohol content for cases of drunk driving. Participants exhale into the CO detector's intake port, and the device generates corresponding data, which is recorded by the researchers. If participants fail to meet the abstinence standard, they will not be eligible for EEG data collection and will need to reschedule their EEG appointment. It is important to note that the CO detected during this experiment will not be preserved or subjected to further testing.

1. ***Drug and equipment management system. [This includes the storage and distribution processes, checking systems, use, and recovery of drugs and equipment]***

This study does not involve the use of drugs or equipment management.

1. ***Data management system. [This includes data collection, management of source data and files, personnel responsible for data collection and entry, and checking systems]***

(1) **Data Collection:** The current study will strictly follow standardized operating procedures for data collection according to different data category.

(2) **Management of Source Data and Files:**

1. The source data is securely stored on the designated data platform. Additionally, multiple backups of the files are created, including non-networked hard drives or external hard drives exclusively used by data analysts.
2. To ensure the security of original data stored on third-party online platforms, timely downloading and secure storage protocols are implemented. This includes measures to enhance platform network security and strengthen terminal protection, preventing any potential information leakage.
3. Regular software and data backups are performed to safeguard against data loss.
4. Vulnerability detection is conducted on a regular basis to identify and address any security weaknesses.
5. Stringent measures are in place to prevent virus intrusions and protect the integrity of the data. By incorporating these revisions, the expression becomes more concise and easier to understand.

(3) The study will designate dedicated personnel responsible for data collection and entry.

(4) **Verification System:** To enhance data quality, a verification system is implemented, which includes a two-person double-checking process and range checks of data values.

1. ***Composition and Responsibilities of the Data Security and Supervision Committee. [Describe the composition of the Data Security and Supervision Committee, including the members' names and contact information. Also, provide information on their roles and responsibilities in ensuring data security and monitoring the study]***

This study did not establish a separate Data Security and Supervision Committee.

1. ***Research Team. [Provide the names of the members of the research team. Describe their roles and responsibilities within the team]***

Project administration: CZF

Experimental design and data analysis: CZF, GQ, CLL, LML

Psychological intervention and training: GJ

Recruitment of participants, data collection, and analysis: CLL, PXY, CZL

1. ***Intellectual Property. [Describe the ownership of intellectual property rights. Explain the policy and order for authorship attribution. Provide information on how the public can access and query the research plan]***

(1) Ownership of Intellectual Property: The trial sponsor.

(2) Authorship Policy and Order: Authorship order will be determined based on the recommendations of the International Committee of Medical Journal Editors (ICMJE).

(3) Access and Time for Public Query of Research Plan: See the publication journal and the China Clinical Trial Registration Center and ResMan Clinical Trial Public Management Platform; 3-5 years.

1. ***Publication Plan. [Provide an estimated timeline and methods for publishing research reports. Specify the number of publications, including interim summary reports if applicable]***

(1) Estimated Time and Method for Research Report Publication: The publication plan will consist of several sections, including the research plan, analysis of panel data using various statistical methods, analysis of psychology-related data, and conclusion.

(2) Estimated Number of Publications: 1 paper (excluding interim summary reports).

1. ***Plan for Sharing Raw Data. [Describe the plan for sharing raw data with the public. Specify the maximum time frame for sharing data, which should not exceed 6 months after the end of the trial. Explain the public platform that will be used for data sharing]***

The original data will be uploaded to the ResMan Clinical Trial Public Management Platform (http://www.medresman.org.cn). The data will be publicly shared no later than 6 months after the end of the trial.

1. ***Treatment and Management of Participants after the Trial Ends. [Describe how participants who have not experienced improvement in their condition at the end of the trial will continue to receive medical care. Explain the measures that will be taken to ensure their ongoing treatment and management]***

In this study, the participants included will be healthy volunteers who will receive guidance from professional teachers in mindfulness training. The goal of this training is to assist participants in effectively managing cravings for smoking by developing skills such as paying attention to and accepting the craving when it arises, and gradually overcoming it. Additionally, this process can enhance the participants' ability to regulate their emotions when confronted with various stressful events in their daily lives.

If, upon completion of the trial, it is determined that the stress level of any participant has reached the clinical threshold, active intervention will be offered by the psychological teachers at the on-campus psychological center. If required, participants will be referred to specialized hospitals for further intervention.

***References***

1. The Writing Committee of 2020 Report on Health Hazards of Smoking in China. 2020 Report on Health Hazards of Smoking in China: an Updated Summary. Chinese Circulation Journal. 2021;36(10):937-52. doi: 10.3969/j.issn.1000-3614.2021.10.001.

2. Jayes L, Britton J, Vardavas C, Leonardi-Bee J. Systematic reviews and meta-analyses on the effects of active and passive smoking on respiratory health outcomes: the SmokeHaz online resource. The Lancet. 2014;384:S42. doi: https://doi.org/10.1016/S0140-6736(14)62168-1.

3. National Center for Chronic Disease Prevention and Health Promotion (US) Office on Smoking and Health. Reports of the Surgeon General. The Health Consequences of Smoking-50 Years of Progress: A Report of the Surgeon General. Atlanta (GA): Centers for Disease Control and Prevention (US); 2014.

4. Global, regional, and national comparative risk assessment of 84 behavioural, environmental and occupational, and metabolic risks or clusters of risks for 195 countries and territories, 1990-2017: a systematic analysis for the Global Burden of Disease Study 2017. Lancet. 2018;392(10159):1923-94. Epub 2018/11/30. doi: 10.1016/s0140-6736(18)32225-6. PubMed PMID: 30496105; PubMed Central PMCID: PMCPmc6227755.

5. Carter BD, Abnet CC, Feskanich D, Freedman ND, Hartge P, Lewis CE, et al. Smoking and mortality--beyond established causes. N Engl J Med. 2015;372(7):631-40. Epub 2015/02/12. doi: 10.1056/NEJMsa1407211. PubMed PMID: 25671255.

6. Piasecki TM. Relapse to smoking. Clin Psychol Rev. 2006;26(2):196-215. Epub 2005/12/15. doi: 10.1016/j.cpr.2005.11.007. PubMed PMID: 16352382.

7. Bu J, Young K, Hong W, Ma R, Song H, Wang Y, et al. Effect of deactivation of activity patterns related to smoking cue reactivity on nicotine addiction. Brain. 2019;142(6):1827-41. doi: 10.1093/brain/awz114. PubMed PMID: 31135053.

8. Sevinc G, Hölzel BK, Greenberg J, Gard T, Brunsch V, Hashmi JA, et al. Strengthened Hippocampal Circuits Underlie Enhanced Retrieval of Extinguished Fear Memories Following Mindfulness Training. Biol Psychiatry. 2019;86(9):693-702. Epub 2019/07/16. doi: 10.1016/j.biopsych.2019.05.017. PubMed PMID: 31303261; PubMed Central PMCID: PMCPmc6788973.

9. Westbrook C, Creswell JD, Tabibnia G, Julson E, Kober H, Tindle HA. Mindful attention reduces neural and self-reported cue-induced craving in smokers. Soc Cogn Affect Neurosci. 2013;8(1):73-84. Epub 2011/11/25. doi: 10.1093/scan/nsr076. PubMed PMID: 22114078; PubMed Central PMCID: PMCPmc3541484.

10. Elwafi H, Witkiewitz K, Mallik S, Thornhill T, Brewer J. Mindfulness training for smoking cessation: moderation of the relationship between craving and cigarette use. Drug Alcohol Depend. 2013;130:222-9. doi: 10.1016/j.drugalcdep.2012.11.015. PubMed PMID: 23265088.

11. Howarth A, Smith JG, Perkins-Porras L, Ussher M. Effects of brief mindfulness-based interventions on health-related outcomes: A systematic review. Mindfulness. 2019;10(10):1957-68. doi: 10.1007/s12671-019-01163-1.

12. Chen Y, Yang X, Wang L, Zhang X. A randomized controlled trial of the effects of brief mindfulness meditation on anxiety symptoms and systolic blood pressure in Chinese nursing students. Nurse Educ Today. 2013;33(10):1166-72. Epub 2012/12/25. doi: 10.1016/j.nedt.2012.11.014. PubMed PMID: 23260618.

13. Bowen S, Marlatt A. Surfing the urge: brief mindfulness-based intervention for college student smokers. Psychol Addict Behav. 2009;23(4):666-71. Epub 2009/12/23. doi: 10.1037/a0017127. PubMed PMID: 20025372.

14. Luberto CM, McLeish AC. The effects of a brief mindfulness exercise on state mindfulness and affective outcomes among adult daily smokers. Addict Behav. 2018;77:73-80.

15. Chan EY. Mindfulness and smoking frequency: An investigation with Australian students. Addictive behaviors reports. 2021;13:100342. Epub 2021/04/01. doi: 10.1016/j.abrep.2021.100342. PubMed PMID: 33786361; PubMed Central PMCID: PMCPmc7988491.

16. Weng X, Luk TT, Lau OS, Suen YN, Lee JJ, Li WH, et al. Brief mindfulness training for smoking cessation in Chinese women in workplaces: A pilot randomized controlled trial. Addict Behav. 2021;113:106677. Epub 2020/10/18. doi: 10.1016/j.addbeh.2020.106677. PubMed PMID: 33069106.

17. Ruscio AC, Muench C, Brede E, Waters AJ. Effect of Brief Mindfulness Practice on Self-Reported Affect, Craving, and Smoking: A Pilot Randomized Controlled Trial Using Ecological Momentary Assessment. Nicotine & tobacco research : official journal of the Society for Research on Nicotine and Tobacco. 2016;18(1):64-73. Epub 2015/04/13. doi: 10.1093/ntr/ntv074. PubMed PMID: 25863520.

18. Gotink RA, Meijboom R, Vernooij MW, Smits M, Hunink MGM. 8-week Mindfulness Based Stress Reduction induces brain changes similar to traditional long-term meditation practice – A systematic review. Brain Cogn. 2016;108:32-41.

19. Zhao X, Chen Z, Kang C, Liu R, Bai J, Cao Y, et al. Mindfulness-based cognitive therapy is associated with distinct resting-state neural patterns in patients with generalized anxiety disorder. Asia-Pacific psychiatry. 2019;11(4):e12368.

20. Gan Q, Ding N, Bi G, Liu R, Zhao X, Zhong J, et al. Enhanced resting-state functional connectivity with decreased amplitude of low-frequency fluctuation (ALFF) of the salience network in mindfulness novices. Front Hum Neurosci. 2022;16:838123.

21. Xiao Q, Zhao X, Bi G, Wu L, Zhang H, Liu R, et al. Alterations of Regional Homogeneity and Functional Connectivity Following Short-Term Mindfulness Meditation in Healthy Volunteers. Front Hum Neurosci. 2019;13:376.

22. Chen S, Fang Y, Chiu H, Fan H, Jin T, Conwell Y. Validation of the nine-item Patient Health Questionnaire to screen for major depression in a Chinese primary care population. Asia-Pacific psychiatry : official journal of the Pacific Rim College of Psychiatrists. 2013;5(2):61-8. Epub 2013/07/17. doi: 10.1111/appy.12063. PubMed PMID: 23857806.

23. Kroenke K, Spitzer RL, Williams JB. The PHQ-9: validity of a brief depression severity measure. J Gen Intern Med. 2001;16(9):606-13. Epub 2001/09/15. doi: 10.1046/j.1525-1497.2001.016009606.x. PubMed PMID: 11556941; PubMed Central PMCID: PMCPmc1495268.

24. Wang W, Bian Q, Zhao Y, Li X, Wang W, Du J, et al. Reliability and validity of the Chinese version of the Patient Health Questionnaire (PHQ-9) in the general population. Gen Hosp Psychiatry. 2014;36(5):539-44. Epub 2014/07/16. doi: 10.1016/j.genhosppsych.2014.05.021. PubMed PMID: 25023953.

25. Spitzer RL, Kroenke K, Williams JB, Löwe B. A brief measure for assessing generalized anxiety disorder: the GAD-7. Arch Intern Med. 2006;166(10):1092-7. Epub 2006/05/24. doi: 10.1001/archinte.166.10.1092. PubMed PMID: 16717171.

26. Lin Q, Bonkano O, Wu K, Liu Q, Ali Ibrahim T, Liu L. The Value of Chinese Version GAD-7 and PHQ-9 to Screen Anxiety and Depression in Chinese Outpatients with Atypical Chest Pain. Ther Clin Risk Manag. 2021;17:423-31. Epub 2021/05/28. doi: 10.2147/tcrm.s305623. PubMed PMID: 34040380; PubMed Central PMCID: PMCPmc8140931.

27. Jiang Y, Wei J. Assessment of the structured clinical interview (SCID) for DSM-5 for somatic symptom disorder in general hospital outpatient clinics in China. 2021;21(1):144. doi: 10.1186/s12888-021-03126-0. PubMed PMID: 33691663.

28. Chen J. The Effects of Transcranial Direct Current Stimulation (tDCS) Combined with Cognitive Intervention on Cigarette Craving and Smoking Volume [Master's Dissertation]: Southwest University; 2020.

29. Tang J, Wang L, Luo T, Wu S, Wu Z, Chen J, et al. Effectiveness of a Brief Mindfulness-Based Intervention of "STOP touching your face" During the COVID-19 Pandemic: a Randomized Controlled Trial. 2022;13(12):3123-33. doi: 10.1007/s12671-022-02019-x. PubMed PMID: 36408118.

30. Liao Y, Wang L, Luo T, Wu S, Wu Z, Chen J, et al. Brief mindfulness-based intervention of 'STOP (Stop, Take a Breath, Observe, Proceed) touching your face': a study protocol of a randomised controlled trial. 2020;10(11):e041364. doi: 10.1136/bmjopen-2020-041364. PubMed PMID: 33234653.

31. Heatherton TF, Kozlowski LT, Frecker RC, Fagerström KO. The Fagerström Test for Nicotine Dependence: a revision of the Fagerström Tolerance Questionnaire. Br J Addict. 1991;86(9):1119-27. Epub 1991/09/01. doi: 10.1111/j.1360-0443.1991.tb01879.x. PubMed PMID: 1932883.

32. National Health and Family Planning Commission of the People's Republic of China. Guideline on China clinical smoking cessation (2015). Chinese Journal of Health Management. 2016;10(2):88-95.

33. Heishman SJ, Singleton EG, Pickworth WB. Reliability and validity of a Short Form of the Tobacco Craving Questionnaire. Nicotine & tobacco research : official journal of the Society for Research on Nicotine and Tobacco. 2008;10(4):643-51. Epub 2008/04/18. doi: 10.1080/14622200801908174. PubMed PMID: 18418787; PubMed Central PMCID: PMCPmc2706572.

34. Li X, Ma R, Pang L, Lv W, Xie Y, Chen Y, et al. Delta coherence in resting-state EEG predicts the reduction in cigarette craving after hypnotic aversion suggestions. Sci Rep. 2017;7(1):2430. Epub 2017/05/27. doi: 10.1038/s41598-017-01373-4. PubMed PMID: 28546584; PubMed Central PMCID: PMCPmc5445086.

35. Cox LS, Tiffany ST, Christen AG. Evaluation of the brief questionnaire of smoking urges (QSU-brief) in laboratory and clinical settings. Nicotine & tobacco research : official journal of the Society for Research on Nicotine and Tobacco. 2001;3(1):7-16. Epub 2001/03/22. doi: 10.1080/14622200020032051. PubMed PMID: 11260806.

36. Yu X, Xiao D, Li B, Liu Y, Wang G, Chen J, et al. Evaluation of the Chinese versions of the Minnesota nicotine withdrawal scale and the questionnaire on smoking urges-brief. Nicotine & tobacco research : official journal of the Society for Research on Nicotine and Tobacco. 2010;12(6):630-4. Epub 2010/05/26. doi: 10.1093/ntr/ntq063. PubMed PMID: 20498226.

37. Deng Y-Q, Liu X-H, Rodriguez MA, Xia C-Y. The Five Facet Mindfulness Questionnaire: Psychometric Properties of the Chinese Version. Mindfulness. 2011;2(2):123-8. doi: 10.1007/s12671-011-0050-9.

38. Yu S, Rodriguez MA, Deng Y, Xiao L, Liu X. The Toronto Mindfulness Scale: Psychometric Properties of the Chinese Version. Mindfulness. 2021;12(8):1976-84. doi: 10.1007/s12671-021-01655-z.

39. Watson D, Clark LA, Tellegen A. Development and validation of brief measures of positive and negative affect: the PANAS scales. JPSP. 1988;54(6):1063-70. doi: 10.1037/0022-3514.54.6.1063. PubMed PMID: MEDLINE:3397865.

40. Huang LY, TingZhong, Ji Z. Applicability of the Positive and Negative Affect Scale in Chinese. Chinese Mental Health Journal. 2003;17(1):54-6.

41. Patton JH, Stanford MS, Barratt ES. Factor structure of the Barratt impulsiveness scale. J Clin Psychol. 1995;51(6):768-74. Epub 1995/11/01. doi: 10.1002/1097-4679(199511)51:6<768::aid-jclp2270510607>3.0.co;2-1. PubMed PMID: 8778124.

42. Yao S, Yang H, Zhu X, Auerbach RP, Abela JR, Pulleyblank RW, et al. An examination of the psychometric properties of the Chinese version of the Barratt Impulsiveness Scale, 11th version in a sample of Chinese adolescents. Percept Mot Skills. 2007;104(3 Pt 2):1169-82. Epub 2007/09/21. doi: 10.2466/pms.104.4.1169-1182. PubMed PMID: 17879649.

43. Edlin JM, Leppanen ML, Fain RJ, Hackländer RP, Hanaver-Torrez SD, Lyle KB. On the use (and misuse?) of the Edinburgh Handedness Inventory. Brain Cogn. 2015;94:44-51. Epub 2015/02/07. doi: 10.1016/j.bandc.2015.01.003. PubMed PMID: 25656540.

44. Yang N, Waddington G, Adams R, Han J. Translation, cultural adaption, and test-retest reliability of Chinese versions of the Edinburgh Handedness Inventory and Waterloo Footedness Questionnaire. Laterality. 2018;23(3):255-73. Epub 2017/08/02. doi: 10.1080/1357650x.2017.1357728. PubMed PMID: 28756732.

45. Qu S, Sheng li. Diagnostic test of screening generalized anxiety disorders in general hospital psychological department with GAD-7. Chinese Mental Health Journal. 2015;29(12):939-44. doi: 10.3969/j.issn.1000-6729.2015.12.010.

46. Pierce C, Block R. Cautionary Note on Reporting Eta-Squared Values from Multifactor ANOVA Designs. Educational and Psychological Measurement - EDUC PSYCHOL MEAS. 2004;64:916-24. doi: 10.1177/0013164404264848.
