# Supplementary material for: Effects of brief mindfulness training on smoking cue-reactivity in tobacco use disorder: Study protocol for a randomized controlled trial
Source: PLoS One. 2024 Apr 22;19(4):e0299797. doi: 10.1371/journal.pone.0299797 (PMC11034654; doi:10.1371/journal.pone.0299797)
Supplement: S3 File — (DOCX) [file pone.0299797.s003.docx]

| **S3 File. All items from the Chinese Clinical Trial Register (ChiCTR) Registration Data Set** | | | |
| --- | --- | --- | --- |
| **Registration number：** | ChiCTR2300069363 | **Registration Status：** | Prospective registration |
| **Date of Last Refreshed on：** | 2023/5/23 | **Date of Registration：** | 2023/3/14 |
| **Public title：** | Brief mindfulness-based intervention on smoking cue responses in nicotine addicts | | |
| **Scientific title：** | Effects of brief mindfulness training on smoking cue-reactivity in Chinese college students with tobacco use disorder: study protocol for a randomized controlled trial | | |
| **Applicant's institution：** | Kunming University of Science and Technology | **Study leader：** | Zhuangfei Chen |
| **Approved by ethic committee：** | Yes | **Approved No. of ethic committee：** | Kmust-MEC-2023-004 |
| **Name of the ethic committee：** | Medical Ethics Committee of Kunming University of Science and Technology | **Date of approved by ethic committee：** | 2023/3/13 |
| **Country：** | China | **Institution** | Kunming University of Science and Technology |
| **Source(s) of funding：** | National Natural Science Foundation of China (No. 32060196) | **Target disease：** | Smoking Addiction |
| **Study type：** | Interventional study | **Study phase：** | Active |
| **Study design：** | Parallel | **Study execute time：** | From 2023-03-04 to 2024-03-04 |
| **Objectives of Study：** | Combined with the subjective report of smoking craving, the moderating effect of "STOP" in mindfulness method on smoking cue response was analyzed by using EEG data. Heart rate and respiration data were added to the result analysis to explore the regulation effect of mindfulness method from a more comprehensive and objective level, so as to provide more research reference for subsequent similar experiments. | | |
| **Inclusion criteria** | 1. Right-handed; 2. Smoking an average of 10 cigarettes (half a pack of cigarettes) per day; 3. Smoking for at least 1 year; 4. Use CO instrument to detect whether it is 10-7ppm or lower; 5. Between 18 and 40 years old; 6. Having normal vision or corrected to normal vision; 7. Normal and healthy mental and physical status (PHQ-9 total score < 20, GAD-7 total score < 11); 8. Met the diagnostic criteria for tobacco use disorder in the DSM-5 (through clinical semi-structured interviews); | | |
| **Exclusion criteria：** | 1. Patients with asthma, contact dermatitis and allergy to silicone gel; 2. Those who have taken steroids in the past 3 months; 3. Practice any meditation practice or yoga, tai chi or qigong for more than 20 hours in the past year or lifetime, attend a meditation or yoga retreat, and attend any meditation course; 4. Not suitable for electroencephalography (such as metal implants and severe cranial injuries); 5. Total score of PHQ-9 >= 20; 6. GAD-7 total score >= 11; 7. Advocating specific religious beliefs, which makes it impossible to meditate according to the needs of the course; 8. Currently participating in similar or other neurophysiological trials. | | |
| **Interventions：** |  | | |
| **Group (Sample size)：** | experimental group (n=45) | control group (n=45) | |
| **Intervention：** | Brief mindfulness training | Relaxation training | |
| **Primary Outcomes：** | Cigarette craving; EEG signal; Heart rate; Breathe; Visual analogue scale, VAS; Five Facet Mindfulness Questionnaire; Average number of cigarettes smoked per day | **Measure time point of outcome：** | -T1, T0 (baseline); T1 (post intervention, i.e., 1-wk), T2-T5 (follow-up; i.e., 1-mon,3-mon, 6-mon,1-year) |
| **Randomization Procedure (please state who generates the random number sequence and by what method)：** | The statistician will assign the experimental group and the control group in a 1:1 ratio according to the order of the participant sequence table. Randomization for this trial will be performed using the random sequence Generator station, https://www.random.org/ | | |
| **Blinding：** | This trial is a group therapy, and the participants are all students in the same school, so it is inevitable that participants who know each other or do not know each other will talk about the contents related to the trial training they received. Blinding the researchers is also challenging, because it is easy to get caught up in the experiment. Therefore, the double-blind requirements of clinical trials cannot be achieved. | **Calculated Results after the Study Completed public access:** | Public |
| **IPD sharing; The way of sharing IPD” (include metadata and protocol, if use web-based public database, please provide the url)** | Yes; http://www.medresman.org.cn | **Data collection and Management (A standard data collection and management system include a CRF and an electronic data capture：** | Raw trial data will be uploaded using the ResMan database, a public platform for clinical research. |
