# Supplementary material for: Effects of brief mindfulness training on smoking cue-reactivity in tobacco use disorder: Study protocol for a randomized controlled trial
Source: PLoS One. 2024 Apr 22;19(4):e0299797. doi: 10.1371/journal.pone.0299797 (PMC11034654; doi:10.1371/journal.pone.0299797)
Supplement: S4 File — (DOCX) [file pone.0299797.s004.docx]

**EEG Preprocessing and Analyses**

EEGlab13.0.0b (https://sccn.ucsd.edu/eeglab) and custom MATLAB R2013b (The MathWorks, Natick, MA, United States) scripts will be used to preprocess the EEG data offline. First, the recorded EEG data will be filtered in the range of 1-48 Hz, followed by a notch filter at 50 Hz; The data will then be re-sampled to 512Hz and epochs of 2 second will be created for each subject. Visual inspection will be used to discard contaminated epochs, and independent component analysis (ICA) will be performed afterward.

After completing preprocessing, subjects who met the following criteria will be excluded: contaminated ICs were more than 5 to be eliminated via the ADJUST plug-in (<https://www.nitrc.org/projects/adjust/>) or bad channel and bad segments with blinking times equal or greater than 10 within 1 min in all data length removed continuously using visual inspection, which was considered as continuous eye-opening state due to participants not follow the instructions. Subsequently, the processed data will be converted into frequency domain representations using the Darbeliai plug-in (v2019.02.01.1; <https://github.com/embar-/eeglab_darbeliai/wiki/0.%20EN>) in EEGlab (<https://sccn.ucsd.edu/eeglab>) to calculate average relative EEG band power at both the single electrode level and ROI level.

For ERP data, the analysis duration (epoch) is 1000ms, with a baseline of 200ms before the presentation of the stimulus. The selected electrodes are F3, FZ, F4 (frontal lobe), FC3, FCZ, FC4 (fronto-central region), C3, CZ, C4 (central region), CP3, CPZ, CP4 (centro-parietal region), and PZ, totaling 13 electrode points. The average wave amplitude of N2 (200-300ms) and P3 (350-550ms) components will be primarily measured and analysed. All the work related to ERP component analysis will be completed by using ERPLAB toolbox.

To compute the microstate topographies, the global field power (GFP), which represents the variance of EEG potential across scalp electrodes, will be calculated for each time point using the MST1.0 EEGlab toolbox [[1](#_ENREF_1), [2](#_ENREF_2)]. The topographies at the peaks of GFP will be extracted for each dataset and further analysed as the best representative topographies across the dataset. The quality of the current cluster assignment is evaluated using the global explained variance (GEV). Subsequently, modified k means clustering will be used to categorize the maps into one of the four model microstate classes (A, B, C, and D). Four microstates will be estimated for the resting-state EEG signals. Regarding the microstate during the cue-reactivity and mindfulness task, we aim to compute the representative microstate that included all task blocks, excluding the remaining resting blocks. This is done to compare the topographies of the microstate models between the resting state and the task. We anticipate obtaining four similar model microstate classes when performing microstate modeling within each condition.

The fieldtrip toolkit will be used to calculate the Phase Lag Index (PLI) [[3](#_ENREF_3)] and Phase Locked Value (PLV) [[4](#_ENREF_4)] for phase synchronized indices.

The PLI is a measure used in neuroscience to quantify the phase synchronization between two signals, specifically the consistency of the phase difference between them. Unlike the Phase Locking Value (PLV), which measures the average phase synchronization, the PLI focuses on the non-zero phase differences only, using formula as follow:

$$PLI=\text{}\text{}\text{sign}\left[ \text{∆}\text{Φ}\left( \text{t}_{\text{k}} \right) \right]\text{}\text{}$$

In here, N is the total number of time points analyzed, ϕ represents the phase difference at each time point, and the sign(ϕ) function returns 1 for positive phase differences and -1 for negative phase differences. TheΣsymbol denotes summation across all the time points. Φ is use mean value and the PLI value range of 0 (no phase locking) to 1(complete synchronization).

The phase locking value (PLV) is defined at time t as the average value of exponential of the phase difference between the two signals:

$${PLV}_{t}=\frac{1}{N}\left| \sum_{n=1}^{N} exp(j\theta(t,n)) \right|$$

N is the total number of time points considered, where θ(t, n) is the phase difference φ_1_(t, n)-φ_2_(t, n). PLV measures the intertrial variability of this phase difference at t: If the phase difference varies little across the trials, PLV is close to 1; it is close to zero otherwise.

A 2-second sliding window will be adopted to calculate the instantaneous phase difference for each sampling point, with a 50% overlap. The frequency bands of θ (4-8 Hz), α (8-13 Hz), low β (13-20 Hz), and high β (20-30 Hz) will be analysed. Both intra-band and cross-band coherence will be calculated. Due to the risk of contamination from electromyographic signals, the γ frequency band will not be included in the analysis. For each frequency band and all participants, n*(n-1)/2 connection values will be calculated by channel/data source. The individual matrices will be averaged and compared between groups to obtain the t-value matrix.

1. Poulsen AT, Pedroni A, Langer N, Hansen LK. Microstate EEGlab toolbox: An introductory guide. bioRxiv. 2018:289850. doi: 10.1101/289850.

2. Tamano R, Ogawa T, Katagiri A, Cai C, Asai T, Kawanabe M. Event-related microstate dynamics represents working memory performance. NeuroImage. 2022;263:119669. Epub 2022/10/08. doi: 10.1016/j.neuroimage.2022.119669. PubMed PMID: 36206941.

3. Stam CJ, Nolte G, Daffertshofer A. Phase lag index: assessment of functional connectivity from multi channel EEG and MEG with diminished bias from common sources. Hum Brain Mapp. 2007;28(11):1178-93. Epub 2007/02/03. doi: 10.1002/hbm.20346. PubMed PMID: 17266107; PubMed Central PMCID: PMC6871367.

4. Lachaux JP, Rodriguez E, Martinerie J, Varela FJ. Measuring phase synchrony in brain signals. Hum Brain Mapp. 1999;8(4):194-208. Epub 2000/01/05. doi: 10.1002/(sici)1097-0193(1999)8:4<194::aid-hbm4>3.0.co;2-c. PubMed PMID: 10619414; PubMed Central PMCID: PMC6873296.
