# Supplementary material for: Effects of brief mindfulness training on smoking cue-reactivity in tobacco use disorder: Study protocol for a randomized controlled trial
Source: PLoS One. 2024 Apr 22;19(4):e0299797. doi: 10.1371/journal.pone.0299797 (PMC11034654; doi:10.1371/journal.pone.0299797)
Supplement: Attachment — Submitted filename: Response to Reviewers.docx [file pone.0299797.s005.docx]

Dear Editor and Reviewers,

We would like to sincerely express our appreciation for the opportunity to address the valuable suggestions. The feedback provided has significantly helped to the improvement of our protocol optimization and the advancement of the research ideas. In response to these insightful suggestions, we have critically reviewed the manuscript, diligently incorporating the recommended revisions, with the aim of enhancing both the overall framework and the finer details. We are pleased to present a comprehensive revision that carefully considers the specific feedback provided. We kindly request the editor and reviewers to evaluate our revised manuscript accordingly.

***Journal requirements:***

*When submitting your revision, we need you to address these additional requirements.*

*1. Please ensure that your manuscript meets PLOS ONE's style requirements, including those for file naming. The PLOS ONE style templates can be found at
https://journals.plos.org/plosone/s/file?id=wjVg/PLOSOne_formatting_sample_main_body.pdf and
https://journals.plos.org/plosone/s/file?id=ba62/PLOSOne_formatting_sample_title_authors_affiliations.pdf*

**Reply**

The manuscript and file names have been edited to comply with PLOS One's style requirements. Please refer to the recently submitted manuscripts.

*2. We note that the grant information you provided in the ‘Funding Information’ and ‘Financial Disclosure’ sections do not match.*

*When you resubmit, please ensure that you provide the correct grant numbers for the awards you received for your study in the ‘Funding Information’ section.*

**Reply**

The grant information in the 'Funding Information' and 'Financial Disclosure' sections has been consolidated. The grant numbers for the awards received for the current study are provided in the 'Funding Information' section.

*3. Thank you for stating the following financial disclosure:
 [This study was supported by the National Natural Science Foundation of China (NSFC) (Nos. 32060196, 82360271 and 82201597) and Yunnan Ten Thousand Talents Plan Young and Elite Talents Project (YNWR-QNBJ-2018-027, YNWR-QNBJ-2018-056), and Innovation team of Stress and disorder in nervous system in Yunnan.].
Please state what role the funders took in the study. If the funders had no role, please state: ""The funders had no role in study design, data collection and analysis, decision to publish, or preparation of the manuscript.""
If this statement is not correct you must amend it as needed.
Please include this amended Role of Funder statement in your cover letter; we will change the online submission form on your behalf.*

**Reply**

The funders had no role in study design, data collection and analysis, decision to publish, or preparation of the manuscript. The financial support was provided in forms of research materials. The statement is included in the cover letter.

*4. We note that you have indicated that data from this study are available upon request. PLOS only allows data to be available upon request if there are legal or ethical restrictions on sharing data publicly. For more information on unacceptable data access restrictions, please see http://journals.plos.org/plosone/s/data-availability#loc-unacceptable-data-access-restrictions.*

*In your revised cover letter, please address the following prompts:*

*a) If there are ethical or legal restrictions on sharing a de-identified data set, please explain them in detail (e.g., data contain potentially sensitive information, data are owned by a third-party organization, etc.) and who has imposed them (e.g., an ethics committee). Please also provide contact information for a data access committee, ethics committee, or other institutional body to which data requests may be sent. Please note that authors, including Corresponding Authors, are not permitted to be the sole point of contact for data requests.*

*b) If there are no restrictions, please provide the minimal anonymized data set necessary to replicate your study findings as either Supporting Information files or to a stable, public repository and provide us with the relevant URLs, DOIs, or accession numbers. For a list of acceptable repositories, please see http://journals.plos.org/plosone/s/data-availability#loc-recommended-repositories.*

*We will update your Data Availability statement on your behalf to reflect the information you provide.*

**Reply**

The statement for data sharing has been revised as “The data generated for this study are available from the corresponding author to researchers who meet the criteria for access to confidential data after the completion of the study” (lines: 532-534).

*5. Your ethics statement should only appear in the Methods section of your manuscript. If your ethics statement is written in any section besides the Methods, please move it to the Methods section and delete it from any other section. Please ensure that your ethics statement is included in your manuscript, as the ethics statement entered into the online submission form will not be published alongside your manuscript.*

**Reply**

The ethics statement in the “status and timeline” subsection has been moved to the subsection of Ethics and dissemination in the Methods section (lines: 578-581).

*6. Please include captions for your Supporting Information files at the end of your manuscript, and update any in-text citations to match accordingly. Please see our Supporting Information guidelines for more information: http://journals.plos.org/plosone/s/supporting-information.*

**Reply**

Captions for tables, figures and additional files have been added at the end of current manuscript, in-text citations were updated.

***Reviewer #1:*** *The design and study aim are good but I would wait to submit until have at least phase 1 data to prove something. Also there should be some reason provided for inclusion and exclusion criteria like why only rt handed etc.*

**Reply**

Thank you for your time and consideration. One purpose of the submission is to seek reviewers' advice to optimize the intervention plan and subsequent experimental settings.

The rationale for recruiting right-handed participants is as follows:

1) This study involves EEG experiments, and there are significant differences in brain activation between different-handed individuals. By recruiting only right-handed participants, we aim to minimize the variability in brain activity patterns and ensure comparability.

2) In the Chinese population, there is a larger proportion of right-handed individuals. Conducting research with right-handed participants facilitates the generalizability of findings within the population.

3) In addition, despite the relatively small sample size in this study, we maintained a balance between left and right hands in terms of keying rules to reduce variability.

*------------------------------*

***Reviewer #2:*** *Thank you for the opportunity to review the manuscript.
The study outlined a research plan aiming to investigate the effects of Brief Mindfulness Training (BMT) on smoking cue reactivity in college students with Tobacco Use Disorder (TUD). A two-arm parallel Randomized Controlled Trial (RCT) design, incorporating multiple data collection time points, various analytical software tools, and rigorous data analysis techniques. Ethical considerations and transparency are emphasized.

Strengths:

Robust Study Design: The choice of a two-arm parallel RCT is well-justified for evaluating the intervention's impact, and the inclusion of multiple data collection time points enhances the study's ability to capture temporal changes.

Comprehensive Data Analysis: The utilization of diverse software tools and detailed analysis plans demonstrates a methodologically rigorous approach. The inclusion of effect size measurements and the commitment to addressing missing data enhance the study's robustness.

Ethical Considerations: The commitment to reporting protocol deviations underscores the study's ethical transparency.

Future Research Directions: The inclusion of prospective avenues for future research provides valuable guidance for further exploration in the field.

Recommendations:

Literature Review: The study would benefit from a more comprehensive literature review to provide context for the research question and to highlight existing knowledge in the area of mindfulness interventions for smoking cessation and cue reactivity. This would help justify the research question and demonstrate how the study contributes to existing knowledge.

Discussion Section: While the discussion section outlines the study's strengths and limitations, it could be improved by providing a more in-depth interpretation of potential implications of the findings. Discussing how the study's results might advance the understanding of smoking cue reactivity and the role of mindfulness in college students with TUD would enhance the discussion's academic value and functionality.

Limitations: While the study acknowledges certain limitations, such as the lack of double-blind methodology and the male-only sample, it would be beneficial to discuss these limitations in more detail and explore their potential impact on the study's outcomes. Additionally, discussing strategies to mitigate these limitations could be valuable.*

**Reply**

Thank you for your invaluable feedback.

We have added content to the literature review section (lines: 130-151), particularly focusing on the application and neural mechanisms of mindfulness-based interventions for nicotine addiction (lines: 171-180; 201-205; 214-223). In this regard, we have provided a comprehensive explanation of how cues reinforce smoking conditioned reflexes and formulated hypotheses based on this process, thereby enhancing the logical reasoning of our research hypothesis. Additionally, supplementary discussions pertaining to potential outcomes have been included (lines: 688-705).

The main outcomes planned for this study focus on multiple aspects, including smoking quantity, subjective craving, and behavioral measurements obtained from the cue-reactivity task (such as reaction time and accuracy). Neurophysiological measures, such as full-band power spectra and coherence indices, will also be assessed. Our specific objective is to investigate neurophysiological indicators that objectively reflect subjective craving reports. By combining behavioral and various neurophysiological measures, we aim to explore the effectiveness of mindfulness intervention in addressing cue-reactivity in smoking and its underlying brain mechanisms. Furthermore, we aim to assess the significance of this treatment approach in targeting psychological dependence in young individuals with smoking addiction (lines: 233-241).

For the potential experimenter bias or participant expectations, the current study will incorporate a three-way evaluation involving participants, psychological teachers, and experimental implementers, which will help partially address this issue (lines:712-716).

*------------------------------*

***Reviewer #3:*** *Given the regional focus, the introduction would benefit from more emphasis on the Healthy China Action, its purpose, aims, and the interventions it aims to accomplish, rather than on global tobacco concerns.*

**Reply**

Thank you for your guidance and expertise.

The purpose, goals, and intervention measures aimed at achieving smoking cessation in the Healthy China Action were elucidated. This includes developing feasible intervention measures at multiple levels, promoting short-term interventions, providing more support to individuals, and emphasizing the importance of the adolescent stage in drug abuse interventions (lines: 112-121).

*The technical descriptions of addiction and cue-induced responses require rewording and clarification. Many sentences appear to exhibit a form of circular logic. For instance, consider the following example: "It is assumed that addiction partially develops due to classical conditioning, where drug-related contextual cues become triggers that automatically prompt drug use." In this context, there is an organism affected by the continued use of a reinforcing stimulus that elicits a strengthened response, commonly referred to as habit strength. Furthermore, the chemical properties of nicotine function on brain pathways in a manner that reinforces negative behavior with continued use. It's worth noting that a cue can be regarded as a stimulus according to basic principles of behaviorism, and "trigger" is essentially another term for cue. Therefore, what is meant by an "automatic prompt" needs clarification. Classic principles can be employed to provide a more scientifically precise description of these concepts.

A thorough explanation of extinction learning is required, including how this process can occur in smokers. Furthermore, it remains unclear how cue-reactivity can be applied beyond the realm of classic stimulus-response terminology.*

**Reply**

A more detailed analysis of the relationship between addiction and cue-induced responses highlights that it is essential to note that the concept of cue-reactivity extends beyond the traditional stimulus-response framework. This complex process involves the integration of various subjective and objective factors (lines: 130-151). The significance of mindfulness as cue exposure paradigms in addiction is also presented accordingly (line 142, 173-180).

*Mindfulness Training (MT) is not widely recognized as a commonly used treatment for Tobacco Use Disorder (TUD) – a citation is needed to support this statement, and any other statements where a claim is made on a fact.*

**Reply**

The sentence has been revised as “mindfulness-based interventions have been explored within the context of treatment for TUD, with promising future in helping to prevent addiction and relapse”, the citation is added accordingly (line: 180).

*Clear content organization is needed to address the organism that encounters a stimulus/cue and how the organism is positively reinforced such that this introduction requires technical scientific concepts and definitions.*

**Reply**

We have expanded the literature review section (lines: 130-151) by particularly focusing on the application and neural mechanisms of mindfulness-based interventions for nicotine addiction (lines: 173-180; 200-205; 214-223). We have provided a detailed explanation of how cues reinforce smoking conditioned reflexes and formulated hypotheses based on this process, thus enhancing the logical reasoning of our research hypothesis. Additionally, we have included supplementary discussions on potential outcomes (lines: 688-705).

The main focus of this study is to examine the anticipated outcomes in several key areas, including smoking quantity, subjective craving, behavioral measures derived from the cue-reactivity task (such as reaction time and accuracy), as well as neurophysiological measures such as full-band power spectra and coherence indices. Our specific objective is to investigate objective neurophysiological indicators that align with subjective craving reports. By integrating behavioral and diverse neurophysiological measures, our aim is to explore the effectiveness of mindfulness intervention in addressing cue-reactivity in smoking and its associated underlying brain mechanisms. Additionally, we aim to evaluate the significance of this treatment approach in addressing psychological dependence among young individuals with smoking addiction (lines: 233-241).

Moreover, We have reviewed researches related to mindfulness that are of priority. Compared to previous studies, incorporating mindfulness exposure may better capture the diverse influences of cue, emotion, and classical conditions on smoking behavior (lines: 175-176).

*The aim of the study to test MT for TUD in young adults adds to the literature that has mainly focused on mid-aged smokers.

METHOD

Terms for measuring urges and cravings are used interchangeably. A subjective report of craving may not suffice as a robust primary outcome. It is advisable to include a task to assess objective resistance to smoking.*

**Reply**

The smoking cue-reactivity picture task has been incorporated, utilizing an existing smoking picture library for task design. This picture library has been validated with multiple published research studies, demonstrating good reliability and validity (lines: 484-488).

*The description of interventions does not provide sufficient details for the interpretation of the independent variable (IV) stimulus. Specific information is needed, such as the duration per day, the delivery modality, whether sessions are conducted individually, and the inclusion of fidelity assessments. It's important to note that the control group does not receive a "relaxation" stimulus; instead, they are merely watching a nature film and unclear how much exposure they get to this film over the 7 days.*

**Reply**

We conducted a research review on the effects and mechanisms of nature documentary films in relaxation training, and we also improved the anticipated recording of relaxation training exercise duration (lines: 420-421).

*Much more detail for the cue reaction task and the scores it will produce. A measure of “brain function” is not technical as stated in the measures.*

**Reply**

The cue-reactivity task and the calculation of brain functional metrics were described in detail in the method and data analysis (lines: 489-499; 610-622; table 2).

*Detailed description of EEG protocol needed and expected scores to be derived with equations.*

**Reply**

We have supplemented the EEG research content and formulas in the data analysis section, and added the referenced literature that serves as the basis for the calculations. The detailed content has been submitted as a supplementary file (S4 File).

*Discussion does not address the major additions to the field expected and what gains will be made.*

**Reply**

We have added additional discussions in relation to the benefits of the study in the form of supplementary discussions (lines: 688-705).
 *Unclear how a RCT design is useful for a 7-day exposure given then complexity of addiction.

Unsubstantiated that a 7-day intervention would have 3, 6, and 12 month effects. This is not a realistic model of addiction treatment and recovery.*

**Reply**

Following the current standard RCT experimental design, we have adjusted it to a multi-session brief mindfulness intervention with each session lasting for one day. We conducted follow-up visits after each session and recorded daily practice time to increase exposure time (lines: 404-410).

*------------------------------*

***Reviewer #4:*** *- Introduction should also have mention of neuropsychiatric changes that might lead to smoking addiction. Smoking cues could be one of the causes for cravings and impulsivity but not the only cause.*

**Reply**

Thank you for your valuable insights and suggestions.

In the background section, we have supplemented a multifactorial explanation for the emergence of craving impulses (lines: 130-151). It emphasizes that cues serve as a dynamic observation window and are an important entry point for investigation (lines: 173-180).

*- No mention of whether participants can be on medication assisted treatment for tobacco use disorder, before or during the study. It's not a bad idea to include that in the exclusion criteria. People do smoke while on MAT. Needs clarification as it can confound the result. Also, it’s not a bad idea to do symptom checklist to identify underlying mental health issues as that could also confound the results. Just a suggestion.
- Number of cigarettes and length of smoking history should be matched in BMT and Control group during analysis*

**Reply**

Participants on medication-assisted treatment for tobacco use disorder will be excluded before or during the study, as added in the exclusion criteria (line: 287).

To ensure demographic matching, participants were required to be matched in terms of smoking severity (i.e., smoking years and daily cigarette consumption), and then randomly assigned to either the BMT or CON group (lines: 355-356).
 *- Secondly, the subjects would all in male, which limited the scope of analysis and 516 generalization.- "the subjects would all BE male"
- Overall, well-designed study and good outcome measures*

**Reply**

The limitations section discussed the influence of gender, emphasizing that hormonal differences can impact brain functional states and potentially limit the generalizability of findings across different gender populations (lines: 717-719).

*------------------------------*

***Reviewer #5:*** *This manuscript describes a study protocol for testing the effect of a brief mindfulness training on smoking cue-reactivity.
The strengths of the study protocol include a RCT design and the level of detail provided about this.
The introduction section was very well written with a good flow; and was extremely detailed offering a great justification for the study. The use of EEG also adds value to the study.*

*I guess my biggest problem with the manuscript is that authors don’t seem to make a case for what this adds to the existing literature. With no results, it is hard to justify the publication of just the protocol.
This would be a much stronger manuscript once the results are available. There could be a case made for publication in the case of a novel protocol that is innovative in some way. This is also especially considering the authors reference multiple other studies in the literature (references 29-34) that have demonstrated effects of brief mindfulness interventions on smoking. Some of the other studies also have similar sample sizes and similar designs. There is value in replication of a study with results, but not without.
Because of the lack of data/results, the manuscript also frequently switches between past and future tense making the language sometimes confusing.
Would also recommended revising the manuscript for grammatical errors and consistency of terminology (example use subjects or participants, not both; preferable use study instead of ‘experiment’).*

**Reply**

Thank you for your insightful recommendations. We have made revisions to the grammar and terminology accordingly. In the discussion section, we have presented prospects for potential outcomes (lines: 656-681,688-705).

The purpose of the submission of the protocol is to seek reviewers' advice to optimize the intervention plan and subsequent experimental settings. Thank you for your valuable suggestions regarding the current protocol’s update.

*Some example of revisions/questions:
Abstract:
- Background paragraph, last line “Try to reveal the dynamic models of brain function underlying the process” is grammatically incorrect.*

**Reply**

The sentencewas revised as follows: “The objective of the present study is to investigate the impact of BMT intervention on smoking cue-reactivity among Chinese college students with tobacco use disorder (TUD). The study aims to uncover the dynamic models of brain function involved in this process.”

*-why are mindfulness measures includes as outcomes measures?*

**Reply**

We hypothesize that multiple short-term interventions can lead to an increase in levels of mindfulness. Through training, individuals in the smoking population can learn new coping strategy to manage cue-induced cravings (both physical and psychological discomfort). Therefore, mindfulness is considered as the primary variable influencing cue reactivity.

*-Please remove* *paranthesis in abstract (Line 66)*

*-indicators reflecting correlation between mindfulness and smoking cue-reactivity ?*

**Reply**

The paranthesis in abstract has been removed.

We will analyze subjective reports of mindfulness, subjective reports of cravings, and corresponding electroencephalographic (EEG) patterns in the same state to explore EEG indices that reflect both mindfulness states and smoking cravings. Regression or machine learning algorithms may be employed for this analysis

*-Overall Study design and participants:
-consider revising line 169— “The subjects meeting the experimental requirements…..”. It’s reads poorly.*

**Reply**

The sentence that “The subjects meeting the experimental requirements will participate in the experiment by the sequence of baseline data collection, intervention training, and data collection of post-intervention as well as follow-up time points.” has been revised as “Participants who meet the experimental requirements will participate in the experiment by following a sequence that includes baseline data collection, intervention training, post-intervention data collection, and follow-up time points” (lines: 257-260).

*-There are multiple references to living environment, explain the significance of this for the study or in the context of smoking behaviours.
Inclusion/exclusion:
- Why is right handedness an inclusion criteria? Why is normal vision one as well ? (These seem related to the cue-reactivity task, but needs to be explained. If left handers were excluded, will that affect the results).*

**Reply**

Handedness has an impact on brain functional activity. Therefore, only right-handed individuals were included to ensure the ability to complete the picture stimulus task accurately, with normal visual acuity, and minimize potential experimental result variations caused by visual differences. The statement that” Handedness and vision criteria ensure task completion and minimize variability” was added in line: 288.

*- the authors also needs to explain exclusion criteria such as contact dermatitis and allergies to silicone.*

**Reply**

Individuals who may have allergies to experimental materials such as conductive gel and electrode adhesive may not be suitable as participants for the electroencephalogram (EEG) procedure during the experiment (lines: 278-279, 282).

*Sample size and power calculation:
- lines 203/204, the sentence is long and run-on with grammatical errors.*

**Reply**

The paragraph has been revised as “In the current study, the primary indicator used to assess smoking urges will be self-reported subjective measures. Based on previous research conducted on similar populations, we have defined a reduction score of 4 on the Brief Questionnaire of Smoking Urges (QSU-B) as the expected decrease in smoking craving. Therefore, in the experimental group after the intervention, we anticipate observing a ΔQSU-B of 4” (lines: 296-300).

*------------------------------*

***Reviewer #6:*** *The manuscript by Cheng et al. studies the important topic of behavioral interventions for the treatment of tobacco use disorder.

Strengths of the paper:
-The title is appropriate for the text's content.
-The introduction to the article is well laid out.
-The methods section clearly describes the study design, subject selection, sample size, etc.
-Discussion further elaborates on the study's objectives and limitations.

Some recommendations:
-Line no. 86: states that health and economic losses amount to "trillions of dollars," which seems to exaggerate the values reported in the study referenced by the authors. I will recommend rephrasing the words to reflect the same.*

**Reply**

Thank you for your mindful suggestion.

The state has been revised as “The health costs and other economic losses caused by smoking had already amounted to trillions of dollars, reaching $1.4 trillion annually a decade ago”. The reference was updated (line: 96).

*-Lines nos. 89 and 96: Need to add* *references.*

*-Line no. 99: The authors state that the National Health Commission of China aims to decrease the smoking rate to 20% by 2030 but did not mention the current smoking rate (or from what value to 20%).*

**Reply**

The references have been supplemented (ref. [8] and [9]).

As To the current smoking rate, we have supplemented the data from the 2020 Report on Health Hazards of Smoking in China, the smoking rate among people over 15 years reach to 26.6% (line: 102).

*-Line no. 148: correct spelling mistake "our previous trail" to trial.
-Line no. 180: Inclusion criteria include "right-handed" and "normal or corrected to normal vision." I will recommend that authors add* ***brief commentary*** *for the rationale of this.*

**Reply**

The spelling mistake in line 148 has been corrected.

To account for the influence of handedness on brain function and activity, the study exclusively includes right-hand individuals. Additionally, a specific criterion for normal vision has been implemented to ensure accurate completion of image stimulation tasks. We have added brief commentary that “Handedness and vision criteria ensure task completion and minimize variability” in line: 288.

*-Line no. 515: Limitations of study state "subjects would all in male." The inclusion criteria do not state this. Also, I will recommend that authors add brief commentary for the rationale of this criteria.*

**Reply**

Discussion on gender limitations has been supplemented (line: 717-719).

*------------------------------*

***Reviewer #7:*** *the paper looks interesting. There are some limitations noticed.

The relationship between EEG results and cravings was not adequately addressed in the study. Additionally, false positive CO was not discussed, and there was very minimal discussion of the cue reactivity task and Oddball Paradigm. The study design had several flaws, including inadequate random sampling, observer bias, and confounders such as lack of blinding, pre-motivated patients, age, affluence, and education level. Furthermore, the specific interventions addressed in the trainings were poorly explained, and there was limited discussion on mindfulness application outside of smoking cessation. In addition, the study's application to the general public and the homogeneity of the Chinese population were not thoroughly discussed.*

**Reply**

Thank you for your constructive suggestion!

1) We appreciate your input regarding the relationship between EEG results and cravings in our study. We provided a review of prior EEG studies and neuroscientific principles related to cravings, including major research directions, and summarized the computations involved. Accordingly, the expected EEG indices are listed in data analysis section (lines: 610-622). The correlation analysis of subjective cravings, behavior and EEG indicators will be used to obtain characteristic brain functional change related to cravings (lines:626-628).
2) Regarding false positives for carbon monoxide (CO), we have added a supplemental discussion in the limitations section (lines:720-722).
3) Regarding the paradigm setup, we have supplemented the information in the methods section (lines: 484-499), also addressed the issues of limitation in randomization and blinding in the discussion section (lines:712-716).
4) We have provided additional elaboration on specific interventions during training (lines:404-414). We have improved the description of daily mindfulness practice (lines: 423-425) and supplemented the significance of the study results for the general population (lines: 692-705). Discussion on paradigm setting and expected objective results were also added (lines:656-681).

Thank you again for your thorough review and valuable feedback! We appreciate your insights and eagerly await any additional suggestions you may have to help us further enhance our protocol.

Best regards,

Zhuangfei Chen

[chen.zhf@outlook.com](mailto:chen.zhf@outlook.com)
