# Supplementary material for: Effects of brief mindfulness training on smoking cue-reactivity in tobacco use disorder: Study protocol for a randomized controlled trial
Source: PLoS One. 2024 Apr 22;19(4):e0299797. doi: 10.1371/journal.pone.0299797 (PMC11034654; doi:10.1371/journal.pone.0299797)
Supplement: Attachment — Submitted filename: Response to Reviewers.docx [file pone.0299797.s006.docx]

Dear Editor and Reviewers,

Thank you for considering our manuscript for revision. We sincerely appreciate all the valuable feedback provided. In response to this feedback, we have carefully revised the manuscript to address the concerns raised and enhance its overall quality to meet the standards required for publication. We are grateful for the opportunity to further improve and strengthen our work. Once again, we thank you for your consideration and valuable input.

***Journal Requirements:***
 *Please review your reference list to ensure that it is complete and correct. If you have cited papers that have been retracted, please include the rationale for doing so in the manuscript text, or remove these references and replace them with relevant current references. Any changes to the reference list should be mentioned in the rebuttal letter that accompanies your revised manuscript. If you need to cite a retracted article, indicate the article’s retracted status in the References list and also include a citation and full reference for the retraction notice.*

**Reply**

We have carefully reviewed the reference list to ensure its accuracy and completeness. We have also added the DOI for each reference article, except for those that were not provided. Thank you for bringing this to our attention and your understanding.

***Additional Editor Comments:***

*Please address concerns raised by reviewer 8, and the article should be ready to accept once these additions are made.*

**Reply**

Thank you for your feedback and suggestion. We appreciate the specific concerns raised by reviewers and we have carefully incorporated the necessary additions to the article to meet the requirements. We appreciate your input and thank you for your time and guidance in improving the quality of our work.

*Reviewer #5: Thank you to the authors for addressing all of the comments. The manuscript is much stronger as a result.*

**Reply**

Thank you for your affirmation and valuable suggestions on enhancing the protocol.

*------------------------------*

*Reviewer #8: This is an interesting study. However there are some fundamental information missing from the manuscript.

Some comments

Is the control group, usual care? As it seems to be another intervention? In which case should this be superiority/non-inferiority design?*

**Reply**

Thank you for your thorough review and valuable suggestions. The study will employ a randomized controlled parallel trial design. In previous mindfulness research, relaxation training has been utilized as an active control (Tang et al., 2016). This training will involves watching documentaries showcasing natural scenery without any additional interventions. It has been suggested that video interventions with relaxing content can have a generic relaxation effect on autonomic regulation (Benz et al., 2022; Connolly, 2004). Hence, the inclusion of relaxation training as an active control in this study follows previous mindfulness research methodologies and enhances the study's superiority design. We have provided a description of the experimental design as follows: "Following previous mindfulness research methodologies, we will integrate relaxation training as an active control in this study to enhance its superiority design" (lines 226-228).

*For the sample size information, its unclear whether this sample size relates to one group, the experimental group or for both, in that is 45 the total?*

**Reply**

Thank you for pointing that out. Our previous statement may have lacked clarity. We have revised this section accordingly. Please refer to lines 279-282, where we clarified that "To ensure proper comparability, an additional 45 participants who match in demographic data and smoking severity will be assigned to the control group. Consequently, a total of 90 participants are required for the present study."

*Define your population of analyses,*

**Reply**

Thank you for your patience in reviewing our work. We have made the necessary supplements regarding the population of analyses as per your suggestion. For both the experimental and control groups, quantitative data, including scale measures and the EEG data, will be collected at baseline, post-intervention and four timepoints in follow-up procedure as mentioned above. All data will be analysed when collection is completed (line 554).

*In the data analysis section, can the authors state that results will be reported in accordance to CONSORT 2010 guidelines.*

**Reply**

Thank you for your suggestion. We have implemented the reporting method specified in the CONSORT 2010 guidelines in the data analysis section. Specifically, we have included the statement "The results of the aforementioned analysis will be reported following the CONSORT 2010 guidelines" (lines 596-597).

*Inclusion of withdrawal of individuals in the study, be more explicit, unless the individuals withdraws consent of use of data, participant data an used at up to the point of withdrawal.*

**Reply**

Thank you for your valuable suggestions. We have made the necessary additions regarding the identification of subject withdrawal validity, as follows: "It is important to emphasize that participant data may be used up to the point of withdrawal, unless individuals explicitly withdraw their consent for data usage." (lines 515-516).

*If there was no data monitoring committee appointed and the trial team reviewed the progress of the trial etc, how was blinding maintained?*

**Reply**

Thank you for your reminder. We have made the following additions regarding the implementation of supervision. "To address the challenges associated with double blinding and the absence of a data monitoring committee, the following procedures are necessary to uphold the integrity of blinding implementation: Impartial assessors will collect and evaluate data without prior knowledge of the study design to ensure unbiased evaluation. The data will be securely managed and analyzed using anonymized coding systems to maintain confidentiality. A clear separation will be maintained between the administrators of the intervention and the assessors of the outcomes to minimize bias. Robust standardized protocols will be implemented to promote consistency and minimize the risk of unblinding. Despite the challenges encountered, these measures are crucial for maintaining the integrity of the study" (lines 681-690).

*Randomisation section, says matched for certain demographics, was this stratified randomisation?*

**Reply**

Yes, stratified randomization will be adopted to ensure a balanced distribution of important variables between the treatment groups, reducing the potential for confounding effects and enhancing the comparability of the groups. By matching participants in terms of demographic data and smoking severity, the study can help control for potential confounding factors that may influence the outcomes.

*------------------------------*

*Reviewer #9: Dr. Lakshit Jain, MD
Minor corrections include:
148: “Our previous trail” looks like the authors are referring to Trial and no trail.*

**Reply**

Thank you for bringing this to our attention. We appreciate the correction, and we sincerely apologize for the spelling mistake in our article. The accurate phrase should indeed be "our previous trial" rather than "our previous trail". We will ensure that this correction is reflected in the revised manuscript. Once again, we apologize for any confusion that may have occurred (line 180).

*Though the study does open the window in a specific direction and provide hypothetical novel approach of assessing brain functioning pre and post BMT and its effects on TUD, did not provide any conclusive data. Further research and data are needed upon conclusion of the study to build up on the findings and address the limitations comprehensively*

**Reply**

We appreciate your positive feedback. Currently, we are still in the protocol stage of our study. We value the input of your suggestion and hope to receive their suggestions to further enhance and refine our research plan through this submission.

*------------------------------*

*Reviewer #10: All the comments seem to be addressed appropriately. Appears to be an interesting study and will be interesting to review results, when finished.*

**Reply**

Thank you for your positive feedback. We appreciate your valuable suggestions for improving the protocol.

We would like to express our sincere appreciation once again for your valuable feedback. Your input has greatly improved the quality and rigor of our study, and we are deeply grateful for your support and valuable suggestions. Thank you for your dedicated efforts in reviewing our work and assisting us in enhancing our research.

Best regards,

Zhuangfei Chen

Email: chen.zhf@outlook.com
